# Adequate emergency department resource usage: Applying simulation-based workshop to improve teaching competence among elementary and junior high school teachers in Taiwan

**DOI:** 10.1097/MD.0000000000027258

**Published:** 2021-09-17

**Authors:** Geng-Shiau Lin, Pei-Ling Tseng, Chia-Chen Chang, Giou-Teng Yiang, Zui-Shen Yen, Jang-Wei Jian, Chen-Yin Tung

**Affiliations:** a Department of Health Promotion and Health Education, College of Education, National Taiwan Normal University, Taipei, Taiwan; b Emergency Medicine Department, Taipei City Hospital, Taipei, Taiwan; c Department of Senior Citizen Service Business, College of Human Ecology and Design, St. John's University, New Taipei, Taiwan; d Emergency Medicine Department, Buddhist Tzu Chi General Hospital, New Taipei, Taiwan; e Emergency Medicine Department, National Taiwan University Hospital, Taipei, Taiwan.

**Keywords:** adequate emergency department resource usage (AEDRU), schoolteachers, simulation-based workshop, teaching competence

## Abstract

**Introduction:**

More than 80% of patients who visited Emergency Department (ED) was not urgent in Taiwan in 2019. It causes insufficient medical services and a latent fiscal threat to the Nation Health Insurance (NHI). This study adopted simulation-based educating modules to explore the effect in teaching competence among primary and middle school teachers for efficient AEDRU (adequate emergency department resource usage) education in the future.

**Method:**

The subjects were 414 elementary and junior high school teachers in Taiwan. 214 participants attended the simulation-based workshop as the simulation-based group, whereas 200 participants took an online self-learning module as the self-learning group. The workshop was created by an expert panel for decreasing the unnecessary usage amount of ED medial resources. The materials are lecture, board games, miniature ED modules, and simulation-based scenarios. A teaching competence questionnaire including ED knowledge, teaching attitude, teaching skills, and teaching self-efficacy was conducted among participants before and after the intervention. Data were analyzed via McNemar, paired t test and the generalized estimating equations (GEE).

**Results:**

The study showed that teachers who participated in the simulation-based workshop had improved more in teaching competence than those who received the online self-learning module. In addition, there were significant differences between the pre-test and post-test among the two groups in teaching competence.

**Conclusion:**

The simulation-based workshop is effective and it should be spread out. When students know how to use ED medical resources properly, they could affect their families. It can help the ED service to be used properly and benefits the finance of the NHI. The health care cost will be managed while also improving health.

## 1 Introduction

Taiwanese citizens or any person working in Taiwan that possesses a National Health Insurance card, has the privilege to access the National Health Insurance program (NHI). It is well known for its cost-effective and comprehensive coverage for quality medical treatment for the Taiwan population and its legal aliens. Over 99% of residents have attended in this system in 2019.^[1]^ However, the quality and medical services have aggravated the abuse of the precious hospital resources. According to government publication, each Taiwanese used medical resources at an Outpatient (OPD) and Emergency Department (ED) an average of 17.3 times in 2019.^[1]^ Moreover, there were 7.12 million the Taiwanese residences who visited the ED once or more in 2018.^[2]^ The overflowing of emergency stations could crowd out not only patients with critical conditions but also build up distrust of the medical system by the general population.^[3,4]^ In fact, there were <2 out of 10 persons who really need immediate medical treatment.^[2]^ Patient dissatisfaction, unintended infections, and risks to patient safety with crowded EDs are a great challenge to healthcare workers.^[5–9]^

This vicious cycle of unnecessary ED visits has also accumulated into an uncontrollable raise in health insurance. It cost 3.9% of the GDP (Gross Domestic Product) in 2020 in Taiwan,^[1]^ and it has become an important topic to manage health care costs while balancing the coverage of health insurance, where properly early education is one of the most effective ways in reversing the abuse of the valuable ED system. Studies of promoting adequate ED resource usage (AEDRU) have been conducted extensively and several health policies have been developed accordingly.^[10–12]^ Most of the studies focused on public health and insurance policies, the improvement of the health care system, and media advertisement resolutions^[13–16]^; but the importance of school education from the perspective of a professional health promotion educator (instead of merely from a health policy perspective) with concepts of AEDRU is uncharted. Since the challenge of NHI cannot be solved by discipline alone, the interdisciplinary cooperation between school education and medicine could be a way forward. Despite individual preliminary knowledge of NHI and ED (i.e., personal experience, information from social news and official propaganda in schools), emergency response and AEDRU are essential topics for elementary and junior high school teachers to incorporate into classroom curriculum. In this way, this study will focus on evaluating the measure in promoting the Education modules for schoolteachers in passing on the AEDRU.

The simulation-based education module has been applied in medical education for years.^[17–19]^ It provides effective learning environments for healthcare workers. With vivid teaching materials and scenarios, participants experience important concepts and practice skills without stress. As simulation-based module applied in surgery, clinical skills, disaster prevention, radiologists, technologists, and nurses training program,^[17–21]^ but it was less seldom used in the lecture of the school teachers. Hence, the miniatures of medical facilities, simulation-based scenarios in ED, and simulation-based AEDRU board games were used in this study. Online education is an alternative solution, especially during the pandemic period. For example, students have been learning from home via online education during COVID-19. In this way, we also designed an online-learning educational module to provide comparison of the effectiveness between the two different modules. The participants were mainly first through ninth grade schoolteachers.

To study the effectiveness of AEDRU education, we selected two observation groups: one was lectured through simulation-based workshops and the other was an online self-learning module. Teaching competence was evaluated through knowledge, attitude, skills, and self-efficacy in a questionnaire. The research objectives are:

1. to assess the effects of the simulation-based learning module in AEDUR teaching competence among schoolteachers;
2. to assess the effects of the online self-learning module in AEDUR teaching competence among schoolteachers; and
3. to discover the effect on the teaching competence between two educational modules.

In addition, we provided the simulation-based material to school teachers as a reference for teaching method in the future. With better understanding of AEDRU, the non-essential visits to the ED from patients can be avoided and lessen the waste of precious NHI resources.

## 2 Methods

### 2.1 Study design and participants

This study adopted a quasi-experimental design and purposive sampling. The participants were elementary and junior high school teachers. The study information was published in a Facebook group, official electronic document, and the notice board of the education departments in different counties and cities in Taiwan. There were 456 participants who volunteered to conduct the study and chose which delivery system they preferred. Excluding the persons who did not complete the questionnaires, 214 teachers chose the workshop as the simulation-based group, whereas 200 teachers took the online self-learning module as the self-learning group. The missing value was 42. The proportions of participants in elementary school (43.5%) and in junior high school (56.5%) were nearly equivalent in both groups.

### 2.2 Simulation-based learning module development and implementation

An expert panel developed a simulation-based learning module which consisted of five professors in health promotion. Six primary and junior school teachers familiar to health promotion education also played a critical role. Finally, four emergency medicine clinical doctors and one ED administrator were also invited to identify and to explain important concepts of ED clinical practices. The panel discussion consolidated the topics within the curriculum of the simulation-based learning module.

Based on teacher empowerment concepts, the simulation-based learning module included a lecture of synergistic integration of the clinical ED principles, board games, medical facility miniatures, and simulation-based scenarios. The process of this module is: first, participants interacted with medical facilities miniatures, and acted out simulation-based scenarios with Q&A in small group activities. Second, the clinical experts explained important concepts within an ED with recent social events and news from public media to help teachers realize the health policy behind reasonable usage of ED resources. Third, the Health Education professors showed these concepts with AEDRU simulation-based board games in small group activities. Last, schoolteachers were encouraged to be more reflective when participating in board games to verify major principles in ED and synthesize information from the simulation-based scenarios that might help them to create curricular activity in schools. Professors also challenged and extended the voluntary teachers’ conceptual understanding and teaching skills by discussing possible educational events in schools during health promotion. At the end of the simulation-based learning module, professors led post-simulation reflection to reinforce key concepts that can stimulate teachers’ future educational activity in schools. The duration of the workshop was four hours, and it was implemented three times in northern, central, and southern Taiwan.

### 2.3 Online self-learning module development and implementation

**Figure 1:**
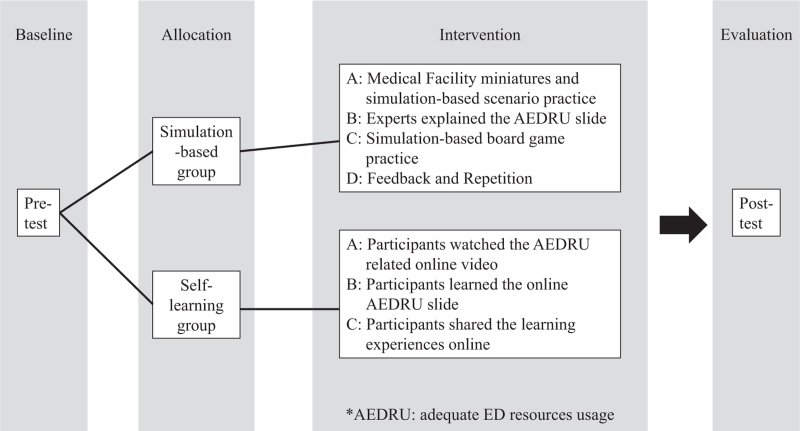
The flowchart for research design and intervention.

The online program offered all information of the workshop including slides and related videos. The link of the online program was offered in the Facebook group or email (if the participants requested). It was available two weeks after all the simulation-based workshops. In the Online module: first, the participants watched AEDRU related videos which covered several ED issues. Then, they watched the slides of ED concepts and the presentation video from the clinical experts on the workshop. Last, they were able to share about their learning experiences online. The flowchart for research design and intervention is shown in Figure 1.

### 2.4 Questionnaires

The questionnaire used in this study was self-reported with close-ended questions. It was divided into ED knowledge (10 questions), teaching attitude (10 questions), teaching skills (7 questions), and teaching self-efficacy (10 questions) in AEDRU. These sections were named “teaching competence.” The content is shown in Table 1. The questionnaire was given before and after the simulation-based health education module or online self-learning module to provide quantitative information.

**Table 1:** Questions in the questionnaire.

Items	Content
ED^∗^ knowledge	
Q1	Which description of ED resources is correct? (1)119 service is one of ED resources. **(2)The functions of ED are receiving, sorting, assessing, stabilizing, and managing patients arriving at its door with different degrees of urgency and complexity.** (3)All ED patients can get treatments in two hours. (4)ED means the express line of Outpatient Department(OPD).
Q2	Which statement is wrong about the difference between OPD and ED? (1)OPD for elective appointments and ED for urgent situations. (2)OPD patients are served on an appointment schedule and ED patients are served on a triage system. **(3)There are more resources in the ED than OPD.** (4)The medical cost in ED is more than OPD.
Q3	The activities in ED include (A)registering (B)clinical evaluation (C)triage (D)discharge or observation. Which sequence is appropriate? (1)ABCD(2)ACBD (3)CBAD**(4)CABD**
Q4	According to the data in Jan. 2018 and Jan. 2019 from National Health Insurance Administration, the proportion of ED urgent cases is. **(1)15%** (2)30% (3)45% (4)60%
Q5	Which of these factors result in inappropriate usage of ED? (A)person medical right (B)less ED doctor (C)ED is 24/7 (D)people won’t be refused using ED resources (E)affordable medical cost **(1)ACDE** (2)ABCD (3)ABDE (4)ABCDE
Q6	What are the adverse impacts of Emergency department overcrowding? (A)patient safety (B)low medical quality (C)high medical cost (D)inadequate healthcare (1)ABC**(2)ABD**(3)BCD (4)ACD
Q7	Which description of an ED triage is WRONG? (1)resuscitation, emergent, urgent, less urgent, not urgent (2)Patients in resuscitation need medical treatment immediately. (3)Patients in emergencies may get treatment in 10 minutes. **(4)Patients in urgent need may get treatment in 30 minutes.**
Q8	Which factors is NOT the benchmark in ED triage? (1)respiration (2)pain (3)consciousness **(4)emotion**
Q9	When you call 119∗, be prepared to answer the call-taker's questions, which may include: (A)location (B)patient's symptom (C)hospital assignment (D) patient's age (1)ABC (2)BCD **(3)ABD** (4)ACD
Q10	Which description of taking an ambulance of 119 service is correct? **(1)people in urgent need won’t be charged.** (2)Patients using 119 services will get priority in ED. (3)Patients may get treatment in the ambulance where they will be well-equipped with advanced medical equipment. (4)The ambulance of 119 services only send patients to the different hospitals in the same city.
Teaching attitude		
Q1	Comprehending concepts related to ED resources is important.
Q2	Positive attitude of AEDRU^∗^ is important.
Q3	Valid skills of AEDRU is important.
Q4	To promote how to use ED resources appropriately at school is important
Q5	It is important to implement the **educational modules** for AEDRU at school.
Q6	It is important to have the **educational materials** for AEDRU at school.
Q7	Educational website for AEDRU at school is important.
Q8	It is important to promote AEDRU at school to increase **students’ knowledge**.
Q9	It is important to promote AEDRU at school to improve **students’ positive attitude**.
Q10	It is important to promote AEDRU at school to develop **students’ skills**.
Teaching skills	
Q1	I can **make the course goals** of AEDRU.
Q2	I can **set the course contents** of AEDRU.
Q3	I can use different educational modules in AEDRU.
Q4	I can use the innovative materials in teaching how to use ED resources appropriately
Q5	I can design the evaluation of teaching outcomes in AEDRU.
Q6	I can create a learning environment in AEDRU.
Q7	I know how to use the website resources to teach AEDRU.
Teaching self-efficacy	
Q1	I can let students realize the characteristics of ED resources via teaching.
Q2	I can help students to avoid misconceptions of using ED resources via teaching.
Q3	I can improve the student empowerment in AEDRU via teaching.
Q4	I can lead students to make the right decision in AEDRU via teaching.
Q5	I can help students to be innovators in AEDRU via teaching.
Q6	I can design multivariate class activities in AEDRU.
Q7	I can control the teaching time in the AEDRU.
Q8	I can make the class atmosphere easy in the AEDRU.
Q9	I can choose the suitable evaluation model for teaching how to use ED resources appropriately.
Q10	I can access the related online information in AEDRU.

∗ 119 = emergency telephone number in Taiwan, AEDRU = adequate ED resources usage, ED = emergency department.

In the ED knowledge section, each correct response was valued as one while incorrect or “don’t know,” responses were scored as 0. Participants were asked to report their subjective teaching attitude to express their opinions in AEDRU, based on a 5-point Likert scale (1 = strongly disagree; 2 = tend to disagree; 3 = neutral; 4 = tend to agree; 5 = strongly agree). The items of teaching skill, and self-efficacy of the participants were rated on a 5-point Likert scale (from 1 = not confident to 5 = very confident).

The questionnaire took ∼15 min to complete. It was reviewed for content validity and reliability by the panel experts, and pilot tested on 20 schoolteachers. The content validity is 0.8 and the Cronbach's α of each item are between 0.68 and 0.96 that shows good reliability. Hence, this questionnaire has been developed appropriately and rigorously.

### 2.5 Statistical analysis

SPSS version 22 (IBM Inc., New York) was used in data analysis among two groups. The ED knowledge section in each group between the pre- and post-test used McNemar to do data analysis because it was categorical and matched-pairs data.^[22]^ The sum of ED knowledge, teaching attitude, teaching skill, teaching self-efficacy sections in each group between the pre- and post-test were analyzed via paired t test due to continuous and matched-pairs data.^[23]^ Moreover, the difference in the pre-test results of the teaching competence was analyzed via independent t test. Because this study adopted purposive sampling, it might have considerable difference in the pre-test results. Thus, we used the generalized estimating equations (GEE) to analyze the differences of teaching competence between different educational modules. GEE is used to estimate the parameters of a generalized linear model with a possible correlation between outcomes. The main advantage of GEE resides in the unbiased estimation of population-averaged regression coefficients despite possible misspecification of the correlation structure. It is applied in repeated measurements, and the correlated responses can be discrete or continuous.^[24]^ For measuring the difference in teaching competence between two groups among intervention, the considering variances in this study were the different timing (pre- or post-test), different group, and the interaction between Time and Group.

### 2.6 Ethics statement

This study was approved by the Institutional Review Board of National Taiwan Normal University in 2018. The Research Ethics Committee number is 201807HS005.

## 3 Results

### 3.1 The effect on AEDRU teaching competence in each group among intervention

Teacher competence was divided into four sections: ED knowledge, teaching attitude, teaching skills and teaching self-efficacy. As shown in Table 2, the sum of scores of all sections in two groups are statistically significant between the pre-test and post-test via pair-*t* test. In the simulation-based group, the average accuracy in knowledge section was 85.19% in the post-test and it was 64.77% in the pre-test; the average score in attitude section was higher in the post-test (4.75) than it was in the pre-test (4.36); the average score in skills section was also higher in the post-test (4.31) than it was in the pre-test (3.65); and the average score in self-efficacy section was also higher in the post-test (4.16) than it was in the pre-test (3.42). There were similar outcomes in the self-learning group. The average accuracy in knowledge section, the average scores in attitude skills and self-efficacy sections were higher in the post-test than they were in the pre-test.

**Table 2:** Comparison of each section in teaching competence by two groups among intervention.

	Simulation-based group (n = 214)			Self-learning group (n = 200)		
Item	Pre-testMean (SD)	Post-test Mean (SD)	*P*	Pre-test Mean (SD)	Post-test Mean (SD)	*P*
ED knowledge (accuracy: %)						
K1	73.33(0.44)	86.67(0.17)	.001	73.50(0.44)	84.50(0.36)	.002
K2	92.38(0.27)	97.14(0.38)	.013	85.50(0.39)	90.50(0.29)	.041
K3	44.29(0.50)	82.86(0.22)	.000	41.00(0.49)	82.50(0.38)	.000
K4	46.19(0.50)	95.24(0.49)	.000	49.50(0.50)	85.00(0.36)	.000
K5	54.76(0.48)	60.95(0.37)	.126	51.50(0.50)	49.00(0.50)	.575
K6	62.38(0.48)	83.33(0.46)	.000	55.50(0.50)	69.00(0.46)	.001
K7	36.19(0.23)	68.57(0.10)	.000	27.50(0.45)	43.00(0.50)	.000
K8	94.29(0.38)	99.05(0.19)	.002	94.50(0.23)	97.00(0.17)	.180
K9	82.38(0.49)	96.19(0.38)	.000	77.00(0.42)	86.50(0.34)	.001
K10	61.90(0.34)	82.38(0.38)	.000	59.00(0.49)	75.50(0.43)	.000
^∗^K	64.77(1.44)	85.19(1.29)	.000	61.45(1.66)	76.25(1.71)	.000
Teaching attitude (score:1–5)						
A1	4.36(0.90)	4.73(0.50)	.000	4.67(0.69)	4.74(0.48)	.131
A2	4.44(0.91)	4.80(0.48)	.000	4.78(0.54)	4.80(0.43)	.697
A3	4.39(0.91)	4.78(0.48)	.000	4.75(0.57)	4.79(0.44)	.333
A4	4.35(0.90)	4.73(0.52)	.000	4.57(0.71)	4.73(0.54)	.000
A5	4.35(0.90)	4.71(0.52)	.000	4.55(0.69)	4.76(0.48)	.000
A6	4.37(0.90)	4.73(0.51)	.000	4.62(0.67)	4.76(0.49)	.001
A7	4.24(0.93)	4.69(0.53)	.000	4.57(0.69)	4.75(0.51)	.000
A8	4.36(0.90)	4.77(0.49)	.000	4.58(0.69)	4.74(0.53)	.000
A9	4.4(0.88)	4.76(0.50)	.000	4.62(0.66)	4.75(0.52)	.005
A10	4.36(0.90)	4.76(0.49)	.000	4.6(0.67)	4.76(0.53)	.000
^∗^A	4.36(0.86)	4.75(0.45)	.000	4.63(0.58)	4.76(0.44)	.001
Teaching skills (score:1–5)						
S1	3.38(0.75)	4.08(0.56)	.000	3.22(0.99)	3.84(0.82)	.000
S2	3.37(0.78)	4.1(0.57)	.000	3.22(0.97)	3.86(0.83)	.000
S3	3.24(0.81)	4.11(0.63)	.000	3.13(1)	3.78(0.88)	.000
S4	3.31(0.84)	4.19(0.58)	.000	3.19(1.02)	3.79(0.91)	.000
S5	3.20(0.79)	4.02(0.63)	.000	3.05(1)	3.73(0.90)	.000
S6	3.36(0.79)	4.11(0.62)	.000	3.19(0.95)	3.85(0.87)	.000
S7	3.5(0.86)	4.23(0.56)	.000	3.41(1)	3.94(0.85)	.000
^∗^S	3.65(0.59)	4.31(0.41)	.000	3.63(0.69)	4.10(0.61)	.000
Teaching self-efficacy (score:1–5)						
E1	3.50(0.74)	4.18(0.56)	.000	3.57(0.94)	4.08(0.78)	.000
E2	3.62(0.71)	4.23(0.57)	.000	3.67(0.97)	4.08(0.77)	.000
E3	3.56(0.75)	4.26(0.57)	.000	3.71(0.89)	4.06(0.79)	.000
E4	3.48(0.74)	4.19(0.59)	.000	3.61(0.95)	4.06(0.79)	.000
E5	3.39(0.77)	4.15(0.61)	.000	3.58(0.96)	4.01(0.79)	.000
E6	3.21(0.81)	4.00(0.66)	.000	3.23(1.07)	3.86(0.87)	.000
E7	3.34(0.77)	4.1(0.60)	.000	3.37(1.02)	3.89(0.82)	.000
E8	3.41(0.74)	4.19(0.59)	.000	3.49(0.96)	3.95(0.82)	.000
E9	3.26(0.80)	4.03(0.63)	.000	3.3(1.03)	3.87(0.87)	.000
E10	3.46(0.79)	4.24(0.58)	.000	3.5(0.96)	3.99(0.88)	.000
^∗^E	3.42(0.66)	4.16(0.50)	.000	3.50(0.86)	3.98(0.73)	.000

A = Teaching attitude, E = Teaching self-efficacy, K = ED knowledge, S = Teaching skills.

∗ an average sum.

To examine the effect on each question in the knowledge section, the McNemar test was used to do data analysis because it was categorical and matched-pairs data. Most of the questions had significant differences between the pre-test and post-test among two groups except question K5 and K8. Participants in the two groups had low accuracy before and after the intervention in question K5. Teachers might confuse the association between less ED doctors and inappropriate usage of ED. Oppositely, both groups got high scores in the pre-test and post-test in question K8 due to the questions easy.

The attitude section was analyzed via pair-t test due to continuous and matched-pairs data. The simulation-based group got higher score in the post-test than in the pre-test in all of questions and there were significant differences between pre-test and post-test in all of questions. Although the self-learning group also got higher scores on the post-test than it did in the pre-test in all of questions, there were no significant differences between pre-test and post-test in question A1, A2, A3 and A9.

The skills and self-efficacy sections was also analyzed via pair-*t* test. Both groups got higher score in the post-test than on the pre-test in all of questions and there were significant differences between pre-test and post-test in all of questions.

### 3.2 The effect on the teaching competence between two groups

Before the intervention, the homogeneity of each section in the teaching competence among two groups was analyzed via independent *t* test. As shown in Table 3, there are statistically significant indicators in knowledge and attitude section but no significant evidence in the other two sections (skills and self-efficacy). Because this study had considerable differences in the pre-test results, we used the generalized estimating equations (GEE) to analyze the differences of teaching competence between different educational modules.

**Table 3:** Comparison of each section in teaching competence by two groups before intervention via independent-*t* test.

Section	Group	N	*M*	SD	df	*t*	*P*
Knowledge	SB	214	0.65	0.14	412.00	2.184	.030
	SL	200	0.61	0.17			
Attitude	SB	214	4.36	0.86	375.31	−3.752	.000
	SL	200	4.63	0.58			
Skills	SB	214	3.65	0.59	412.00	0.368	.713
	SL	200	3.63	0.69			
Self-efficacy	SB	214	3.42	0.66	371.18	−1.034	.302
	SL	200	3.50	0.86			

SB = Simulation-based group, SL = Self-learning group.

To examine the effect on the teaching competence, all of the sections were statistically significant (Table 4). “Time” (the reference time is pre-test), “Group” (the reference group is the self-learning group), and the interaction- “Time∗Group” were the considering variances used to measure the difference in teaching competence between two groups among intervention. As shown in Table 4, “Time∗Group” showed that there was a significant difference (*P* < .001) in ED knowledge and the accuracy of simulation-based group was improved by 0.056 more than that of the self-learning group after the intervention. There was also a significant difference in teaching attitude (*P* < .001), teaching skills (*P* < .001) and teaching self-efficacy (*P* < .001). Hence, the score of the simulation-based group was increased by 0.26 more than that of the self-learning group after the intervention in teaching attitude. It was increased by 0.18 more in the teaching skills and by 0.25 more in the teaching self-efficacy. The score range in attitude, skills and self-efficacy was 1 to 5.

**Table 4:** The effect on the teaching competence via GEE analysis.

Item	Estimate	Std. Error	95%CI^∗^	*P*
ED knowledge				
Intercept	6.15	0.012	0.592∼0.637	<.001
Time (ref. = pre-test)	0.148	0.012	0.125∼0.171	<.001
Group (ref. = self-learning group)	0.033	0.015	0.003∼0.63	.029
Time^∗^Group	0.056	0.017	0.23∼0.89	<.001
Teaching attitude				
Intercept	4.63	0.04	4.55∼4.71	<.001
Time (ref. = pre-test)	0.13	0.04	0.05∼0.2	<.001
Group (ref. = self-learning group)	−0.27	0.07	−0.41 to −0.13	<.001
Time^∗^Group	0.26	0.07	0.12∼0.4	<.001
Teaching skills				
Intercept	3.63	0.05	3.53∼3.72	<.001
Time (ref. = pre-test)	0.48	0.04	0.41∼0.55	<.001
Group (ref. = self-learning group)	0.02	0.06	−0.1 to 0.15	.71
Time^∗^Group	0.18	0.05	0.08∼0.29	<.001
Teaching self-efficacy				
Intercept	3.50	0.06	3.38∼3.62	<.001
Time (ref. = pre-test)	0.48	0.04	0.4∼0.57	<.001
Group (ref. = self-learning group)	−0.08	0.07	−0.22 to 0.07	.29
Time^∗^Group	0.25	0.06	0.13∼0.37	<.001

∗ CI = confidence interval.

**Figure 2:**
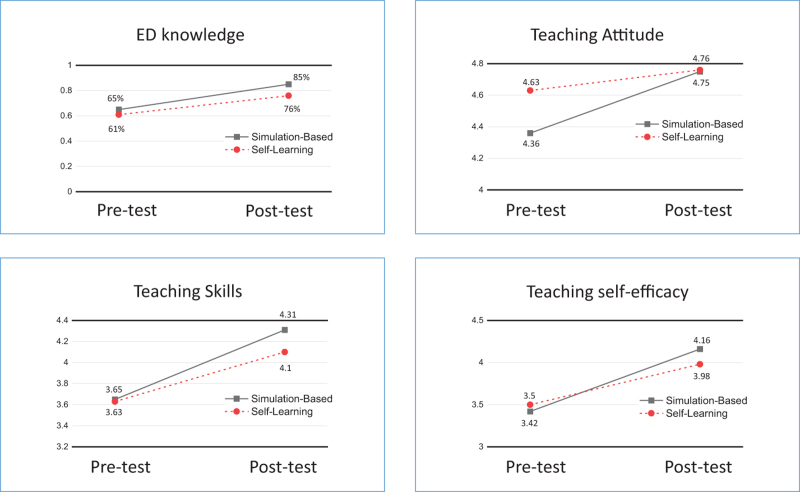
Comparison of the teaching competence by two groups among intervention via GEE.

In addition, all of GEE analysis graphics showed that the slope of the simulation-based group is bigger than the self-learning group. The detail was presented in Figure 2. Therefore, the participants in the simulation-based module improved more than those in the online self-learning module in teaching competence.

## 4 Discussion

As Dr Himmelstein and Dr Woolhandler indicated, 530,000 American families (67%) suffer bankruptcies each year that are linked to illness or medical bills.^[25]^ People impoverished by the huge medical debt continue to issue great challenge to modern society.^[26,27]^ Although NHI in Taiwan is more than insurance and provides healthcare within affordable payment,^[28]^ the financial situation of NHI has become worse since 2004.^[29]^ Inappropriate ED usage is one of the major challenges for NHI.

Education might provide possible solutions but there are few related studies in Taiwan or other countries.^[30,31]^ Considering the willingness, availability, knowledge of health promotion, and teaching skills of health promotion educators, factors appear to be well suited to fulfill supportive roles of the promotion of AEDRU at schools. Knowledge, attitude, and practice (KAP) surveys have been widely used to gather information in many subjects such as medicine,^[32]^ public health,^[33]^ exercise science,^[34]^ economy,^[35]^ and ecology ^[36]^ for years. It provides objective measurement of health education activities in changing health-related behaviors. Therefore, it was applied to this study to provide objective assessment.

Several courses regarding medical issues have adopted simulation-based education to connect the real situations over the years.^[37]^ The simulation-based learning module induces participants’ learning from operation via exposure, sequence, feedback, and repetition.^[37]^ It is able to help participants improve more in knowledge, attitude and skills than those within a traditional learning module.^[38,39]^ Piryani et al developed a one-day simulation-based workshop for 17 health educators to evaluate the perceptions of participants on simulation-based education and a simulation-based workshop. They used a questionnaire for measurement. The results showed that the simulation-based education was not only able to improve the doctor–patient communication skills but also enhance the ability in care and medical issues.^[38]^ Another study was focused on 1178 medical students to process simulation-based education. The results pointed out that simulation-based education could improve the reflective capacity and communication of students.^[39]^ The simulation-based learning module in this study also presented the positive effectiveness among schoolteachers in AEDRU.

In this study, we received positive feedback that many teachers expressed greater willingness to continue this topic as part of the health promotion course in school. Teaching competence is all improved in the ED knowledge, teaching attitude, teaching skills and teaching self-efficacy. The cumulative self-efficacy and KAP scores were higher in the simulation-based group compared to the self-learning groups.

Teachers participated in board games with simulation-based scenarios which included important concepts from different professions. The board game designed as the teaching material could encourage the teacher to imagine what the teaching activity will be like, and to engage challenges they might have in class. Teachers who were involved in the simulation-based learning module could use the teaching materials during real-time classroom interactions to a greater degree than those who learned from the online self-learning module.

During the study period, teachers all showed positive attitudes towards AEDRU, and they all demonstrated positive teaching attitudes in practicing the board games and scenarios in schools. Compared with the online self-learning module, the simulation-based learning module not only provides teachers medical clinical concepts, but also emphasizes group interaction and discussion. Teachers could clarify content knowledge with clinical medical experts and learn teaching skills through interaction and discussion in the workshops.

In summary, teaching competence in AEDRU improved substantially after the intervention. The simulation-based learning module is also able to enhance teacher empowerment. Teachers were encouraged to form an online community for discussion of practices and to be collaborative and especially reflective in their professional health promotion activity. The sharing of the resources that the teachers themselves created is also essential in bolstering efforts in effective lesson planning.

## 5 Conclusions

The simulation-based learning module proves effective and it should be spread. When students know how to use ED medical resources properly, their families will also benefit. Health care cost will decrease while also improving health outcomes if used correctly.

Health education in elementary and junior high schools in appropriate use of ED medical resources is important. Teachers’ perceptions of the knowledge, teaching attitude, teaching skills, and self-efficacy were critical for their adoption of utilizing ED access. Through hands on learning experiences, teachers were able to see many facets of the simulation-based health education module and build their own understanding of the characteristics of the curriculum innovation at schools.

Through using the board game, ED miniature, and simulation-based scenarios, we recognized the key factor that leads to the success of accessing ED use reasonably, is the teaching activity of the teachers. We look forward to the participants playing an important role in the process of diffusion of AEDRU. They will apply the materials from the simulation-based learning module in class activities, and develop similar but innovative education modules in AEDRU to solicit the students’ interest and attention. The promotion by and feedback from teachers are critical in future series research.

## Author contributions

**Conceptualization:** Geng-Shiau Lin, Pei-Ling Tseng, Chia-Chen Chang, Giou-Teng Yiang, Zui-Shen Yen, Chen-Yin Tung.

**Data curation:** Geng-Shiau Lin, Pei-Ling Tseng, Chia-Chen Chang, Jang-Wei Jian.

**Formal analysis:** Geng-Shiau Lin, Pei-Ling Tseng, Jang-Wei Jian.

**Investigation:** Geng-Shiau Lin, Chia-Chen Chang, Chen-Yin Tung.

**Methodology:** Geng-Shiau Lin, Chia-Chen Chang, Chen-Yin Tung.

**Project administration:** Geng-Shiau Lin, Chia-Chen Chang, Giou-Teng Yiang, Zui-Shen Yen, Chen-Yin Tung.

**Resources:** Geng-Shiau Lin, Chen-Yin Tung.

**Supervision:** Geng-Shiau Lin, Chen-Yin Tung.

**Validation:** Geng-Shiau Lin, Pei-Ling Tseng, Giou-Teng Yiang, Zui-Shen Yen, Chen-Yin Tung.

**Visualization:** Geng-Shiau Lin, Pei-Ling Tseng, Giou-Teng Yiang, Zui-Shen Yen, Chen-Yin Tung.

**Writing – original draft:** Geng-Shiau Lin, Pei-Ling Tseng.

**Writing – review & editing:** Pei-Ling Tseng, Chen-Yin Tung.
